# The economic impact of clinical pharmacists in a tertiary care centre in Germany: pharmacoeconomic benefit calculation using cost-avoidance analysis

**DOI:** 10.1186/s12913-026-14527-w

**Published:** 2026-04-25

**Authors:** Julia Borella, Donald Ranft, Dominik Rottenkolber, Nikolaus von Dercks, Thilo Bertsche, Yvonne Remane

**Affiliations:** 1 Pharmacy, University of Leipzig Medical Centre and Leipzig University Medical Faculty, Leipzig, Germany; 2 Drug Safety Centre, Leipzig University and University of Leipzig Medical Centre, Leipzig, Germany; 3 Alice-Salomon-Hochschule Berlin, Department II – Health & Education, University of Applied Sciences, Berlin, Germany; 4 Department of Medical Management, University of Leipzig Medical Centre, Leipzig, Germany; 5 Clinical Pharmacy, Institute of Pharmacy, Medical Faculty, Leipzig University, Leipzig, Germany

**Keywords:** Cost savings, Economics, Pharmacy service, Hospital, Pharmacists

## Abstract

**Objective:**

Pharmacoeconomic evaluation of clinical pharmacy services (CPS) aims to assess the contribution of clinical pharmacists (CP) to optimize the medication process by interventions while simultaneously saving costs. The objective of this study was to provide a valuable contribution by calculating the pharmacoeconomic benefit per full-time equivalent (FTE) comparing costs of avoided adverse drug events (ADE) with CP wage costs in a German tertiary care centre.

**Methods:**

CPs recorded their interventions according to an internal documentation tool, and an expert panel evaluated the likelihood that these interventions would have averted an ADE. The internal cost-avoidance analysis involved determining the total amount of ADE excess costs that could potentially be avoided in 2021. This was achieved by multiplying the median probability of an ADE occurring, as evaluated by an expert panel, by the inflation-adjusted ADE excess costs. These factors, in combination with CP wage costs contributed to the calculation of the pharmacoeconomic benefit. FTEs were used to improve comparability. Deterministic sensitivity analysis was conducted for relevant parameters.

**Results:**

Some 221 out of 3,562 recorded active interventions were analysed. The major interventions made by CPs were medication stops or dosage changes, representing more than 50% of all CPS provided in 2021. To sum up, the findings demonstrated that interventions preventing ADEs are beneficial. A pharmacoeconomic benefit of €13,467 per FTE was calculated. The sensitivity analysis revealed further savings potential, as our model was based on rather conservative assumptions.

**Conclusion:**

This study has demonstrated pharmacoeconomic benefits of CP interventions. The necessity of integrating CPs into routine care to improve patient care quality while increasing economic efficiency has been proven. Future studies should investigate the long-term effects of these interventions and their implementation in other healthcare facilities to determine the full potential of CPs.

## Abstract (de)

***Strengths and limitations of this study***

• Added value by clinical pharmacists

• Real-world data based on continuous documentation

• Evaluation by an expert panel

• Retrospective single-centre study

• Hospital perspective

## Introduction

Although clinical pharmacy services (CPS) have been shown to improve the quality of care and reduce mortality, they are currently not widely implemented as a standard of inpatient care in German hospitals. However, their primary objective extends beyond improving pharmaceutical care; they are also expected to generate economic benefits. Given limited budgets and the economic constraints resulting from diagnosis-related group (DRG)-based reimbursement in Germany, it is crucial to carefully evaluate the benefits of clinical pharmacists (CP) as additional staff members.

International studies reveal that CP contribute to improving patient care and reducing healthcare costs through various measures [1]. They play a key role in preventing adverse drug events (ADEs), identifying and resolving drug interactions, and optimizing drug dosages [2–4]. Their high level of expertise enables them to ensure that patients’ drug therapy is not only effective, but also safe and cost-efficient. Despite the demonstrated benefits of CP-based interventions for patient care, appropriate evaluation tools for the economic implications of these interventions have rarely been applied in Germany. International evaluation approaches are based on different key areas such as adverse drug reactions [5], medication errors, and medication-related adverse drug events [6]. Common cost-avoidance models assess the savings potential of CP interventions in preventing ADEs [7]. Hence, we use the term ADE throughout our documentation and calculation.

In response to the absence of specific costing models for CPS in the literature, the aim of this study was to provide a valuable contribution by calculating the annual avoided ADE excess costs per full-time equivalent (FTE) from the hospital’s perspective. According to a preliminary study [8], our estimated avoided costs refer to the direct hospital treatment costs and do not include broader societal costs or public economic aspects.

## Methods

**Fig. 1:**
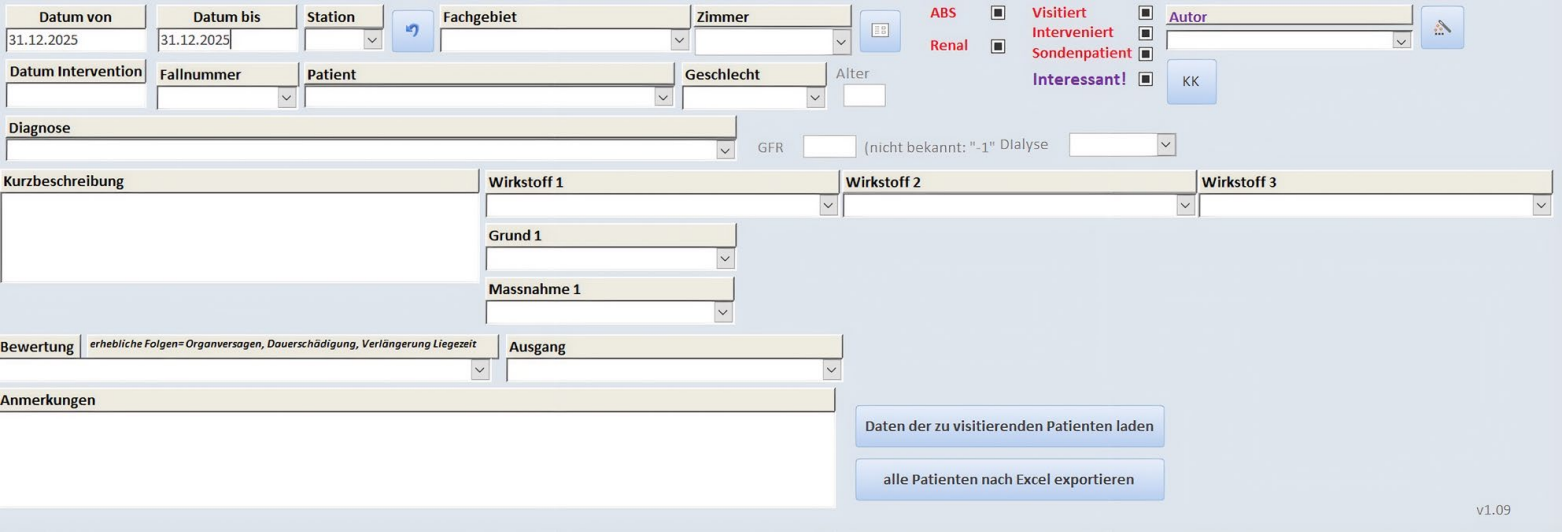
Internal documentation tool for CP interventions. *Caption: As the internal documentation tool is in German language the translation is provided as follows: Datum=date*,* Diagnose=diagnosis*,* Kurzbeschreibung=short description*,* Wirkstoff=drug*,* Maßnahme=intervention type*,* Grund=reason*,* Bewertung=evaluation*,* Ausgang=acceptance*,* visitiert=visited*,* interveniert=intervened*,* Anmerkungen=additional remarks*

Various different documentation tools exist for the purposes of tracking CPS that have been performed. Due to the high effort involved in documenting CP interventions, we applied a simplified internal documentation tool based on Microsoft Access and Excel 2010 for a more convenient daily use (Fig. 1).

This continuous documentation contained the affected drug, short description of the intervention, as well as the reason for the intervention, intervention type, its evaluationand the acceptance. Furthermore, diagnosis and additional remarks could be recorded. Up to three different drugs could be linked to one CP intervention (e.g., due to interaction potential or contraindications). If medication changes such as substituting atorvastatin instead of simvastatin in combination with amlodipine was implemented, the intervention was considered as accepted by medical staff. Adult patients in 2021 were retrospectively analysed in this study, only.

The documentation tool was completed by our CPs (5.75 FTE), who have up to 14 years of professional experience as CPs in a German tertiary care centre. In 2021, these CPs completed 20,972 documentation records.

For our analysis, only patients with a recommended intervention were included (7,399). Out of these, 3,907 interventions were accepted by medical staff, with 3,562 interventions involving concrete actions capable of generating cost savings. Interventions limited to information sharing without further action (345/3,907) were excluded. The remaining 3,562 interventions formed the basis for our pharmacoeconomic analysis, enabling a detailed assessment of the pharmacoeconomic benefit of CP involvement. The intervention types were analysed in terms of content and statistics.

**Fig. 2:**
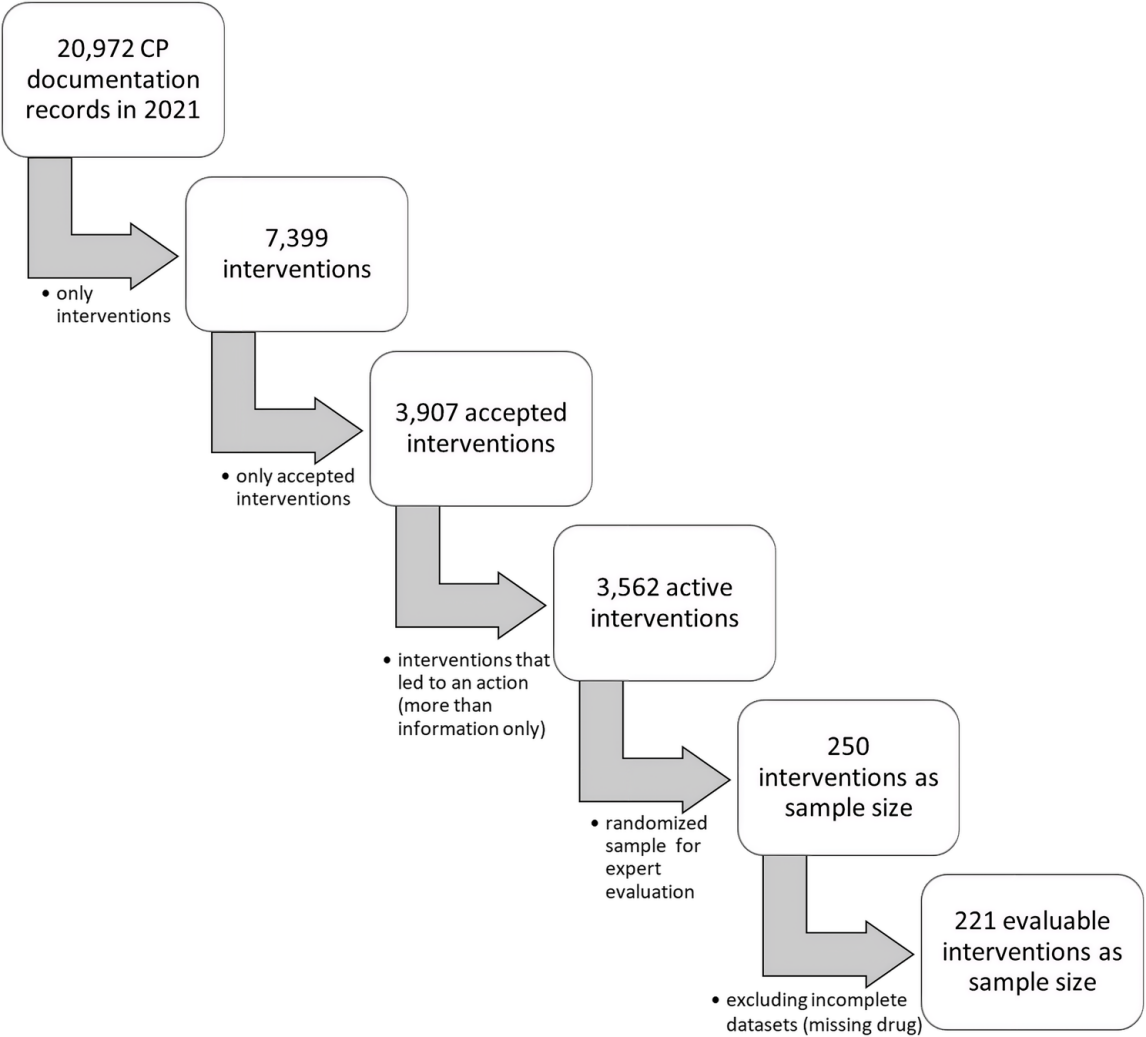
Data extraction process from internal documentation tool

For our expert panel evaluation, a randomized sample (250 out of 3,562 interventions) was created via Microsoft Excel 2010. In total, 29 records were excluded due to missing drug information resulting in 221 evaluable interventions (Fig. 2).

The expert panel consisted of six specialists, four pharmacists (comprising two internal and two external senior CPs with > 5 years of experience), and two hospital senior physicians (consultants in anaesthesiology with > 10 years of experience). Their task was to assess the ADE probability occurrence score using a predefined scale [0.0, 0.01, 0.05, 0.1, 0.25, and 0.5] on the basis of a methodology described by Nesbit et al. [9] and further refined by Patanwala et al. [10]. The evaluation was performed under the assumption that an ADE might have occurred without a CP intervention.

Based on a literature review (PubMed, Embase) and existing approaches to terminology, data documentation, and pharmacoeconomic evaluation of CPS, we developed our calculation model. This model contains FTE wage cost and avoided ADE excess costs over a defined period. The overall result was stated as pharmacoeconomic benefit, meaning a net benefit observed for CPs performing interventions to prevent ADEs per FTE in 2021.

The pharmacoeconomic benefit was calculated as follows (Eq. 1):

1 $$\begin{gathered}\:Pharmacoeconomic\:benefit = \hfill \\\:\:\frac{\begin{gathered}\left[ {\sum \: _{i = 1}^n\left( \begin{gathered} media{n_{ADE\:probability\:occurence\:scor{e_i}}} \hfill \\\times \:avoided\:ADE\:excess\:cost{s_{inflation - adjusted}} \hfill \\ \end{gathered} \right)} \right] \hfill \\\times \:\frac{{basic\:population\:\left( N \right)}}{{sample\:size\:\left( n \right)}}\: - \:\:FTE\:wage\:costs \hfill \\ \end{gathered} }{{FTEs}} \hfill \\ \end{gathered}$$

Eq. 1: Calculation of pharmacoeconomic benefit

The ADE probability occurrence score was evaluated by the six experts, who meticulously examined the 221 interventions regarding probable ADEs. The evaluation was conducted in accordance with the Nesbit/Patanwala scale [9, 10]. The median per intervention was utilized for subsequent calculation. First, the median ADE probability occurrence score for each intervention of the sample was multiplied by the avoided ADE excess costs. The ADE excess costs (e.g. those incurred by a prolonged hospital stay or additional treatment as per the evaluations of the expert panel) were taken into account.

Second, this sum was multiplied by basic population (N) over the sample (n). The FTE wage costs were subsequently deducted. Finally, this result was divided by the number of FTEs calculating the pharmacoeconomic benefit per FTE.

The variables are explained in Table 1:

**Table 1:** Description of equation variables of pharmacoeconomic benefit

Variable	Description	Source
$$\:{median}_{\begin{array}{c}ADE\:probability\:\\\:occurence\:score\end{array}}$$	Contains the median of ADE probabilities evaluated by an expert panel consisting of six specialists. Their task was to assess the ADE probability occurrence score [0.0, 0.01, 0.05, 0.1, 0.25, and 0.5] to the assumption that an ADE might have occurred without a CP intervention. We used the median in our formula to outweigh outliers.	[9, 10]
$$\:{\begin{array}{c}avoided\:ADE\:\\\:excess\:costs\end{array}}_{\begin{array}{c}inflation-\\\:adjusted\end{array}}$$	This study is based on a preliminary research paper published in 2012 on the ADE excess costs in German hospitals. It was adjusted to medical inflation rather than general price increase to reflect healthcare specific inflation.	[8, 11]
$\:basic\:population\:\left(N\right)$	All CP interventions in 2021, that led to an action.	own data
$\:sample\:size\:\left(n\right)$	Random sample of 221 out of 3,562 CP evaluable interventions in 2021.	own data
$\:FTE\:{wage\:costs}_{}$	Wage costs for 5.75 CPs in 2021, assuming 70% -time allocation for the interventions.	own data
$\:FTEs$	Full time equivalents of CPs in 2021.	own data

As all costs and outcomes occurred within the same year, discounting was not applied. Additionally, a deterministic sensitivity analysis using Microsoft Excel 2016 was conducted accounting for uncertainty in the key parameters. Parameters are ranked by the absolute range of variation in pharmacoeconomic benefit in comparison to the base value. Therefore, we used different variables (e.g. ADE excess costs [6, 8]) testing the change in our outcome. Inflation-adjustment of 2.5% was chosen for adapting ADE excess costs to 2021 to reflect sector-specific price dynamics [11]. A conservative comparison analysis showed the general inflation rate in Germany during the same period (1.4%), known as Consumer Price Index [12]. Sectoral health inflation scenarios of 5% per annum were based on observed price increases in the health sector and international projections to evaluate the impact of above-average price developments. The analytic time horizon was one year. The range of ADE probabilities was determined by established models [9, 10]. FTE wage costs for 2021 were declared as the minimum, wage increases of 10% representing the maximum. Finally, a best-case and worst-case scenario was developed. The minimum and maximum values of the corresponding variables were used simultaneously to determine the overall pharmacoeconomic benefit. The reporting of the results followed the Consolidated Health Economic Evaluation Reporting Standards 2022 (CHEERS 2022) [13].

## Results

Firstly, a brief evaluation of the internal documentation tool reveals 20,972 documented CP visits in 2021, resulting in 3,562 (17%) active interventions. More than 50% of all 3,562 active interventions carried out by CPs represent medication stops and dosage changes. An abundant quarter (28.1%) pertained to the recommendations or changes of medication occurring in 10.9%. Laboratory control was seldom requested (3.3%), and recommendations from other experts, such as interactions with the nutrition team were similarly rare counsellors (1.4%). Examples of each intervention type and their relative frequencies are illustrated in Table 2.

**Table 2:** Intervention types with examples and relative frequencies in 2021

Intervention type	Example	Relative Frequency [%]
medication stop	no (more) indication given for pantoprazole	27.7
dosage change	weight adjustment of tinzaparin dose	26.3
new medication recommendation	macrogol for obstipation prevention	15.5
medication change	atorvastatin instead of simvastatin in combination with amlodipine	12.6
physician information	potassium substitution due to hypokalaemia	10.9
laboratory control request	determine valley level of vancomycin	3.3
counselling recommendation	nutrition team recommendation to start liquid nourishment	1.4
nursing staff information	different intake times because of interaction between aspirin and metamizole	1.3
drug form change	switching to oral antibiotics like linezolid	0.8
application modification	counsel regarding alendronate administration	0.05
patient information	explain necessity of calcium having osteoporosis	0.05

In 2021, more than half (52.6%) of the active interventions were successfully implemented. Their permanence remains unclear, as there was no follow-up report.

The expert panel evaluation revealed that most events occurred within a low ADE probability range. However, the contributions were comparable to the proportion of avoided ADE excess costs, despite the occurrence of a small number of high-cost events. Neither the lowest ADE probability score [0], nor the highest one [0.5] have been chosen by all experts simultaneously during their evaluation. Furthermore, the product of the median ADE probability occurrence score for each intervention of the sample and the associated ADE excess costs increased with higher probabilities of an ADE occurring without the intervention.

Table 3 contains the basis data for pharmacoeconomic benefit calculation.

**Table 3:** Data for pharmacoeconomic benefit calculation

Variable	Data
$$\:{median}_{\begin{array}{c}ADE\:probability\:\\\:occurence\:score\end{array}}$$	Specific median calculated based on the expert panel evaluation for each intervention (data not shown)
$$\:{\begin{array}{c}avoided\:ADE\:\\\:excess\:costs\end{array}}_{\begin{array}{c}inflation-\\\:adjusted\end{array}}$$	€970 in 2012 2.5% inflation, equals €1,211 in 2021
$\:{\sum\:}_{i=1}^{n}\left({median}_{A{DE\:probability\:occurence\:score}_{i}}\times\:{avoided\:ADE\:excess\:costs}_{inflation-adjusted}\right)$	€444,386
$\:basic\:population\:\left(N\right)$	3,562
$\:sample\:size\:\left(n\right)$	221
$\:FTE\:wage\:costs$	€366,951
$\:FTEs$	5.75

This results in a pharmacoeconomic net benefit of €13,467 (Eq. 2).

2 $$\begin{gathered}\:Pharmacoeconomic\:benefit = \: \hfill \\\frac{\begin{gathered}\left[ {\sum \: _{i = 1}^{n = 221}\left( {media{n_{ADE\:probabilitiy\:scor{e_i}}} \times \:{\mathrm{\texteuro}{1,211}}} \right)} \right] \hfill \\\times \:\frac{{{\mathrm{3,562}}}}{{221}}\: - {\mathrm{\texteuro}{366,951}} \hfill \\ \end{gathered} }{{5.75}} \hfill \\= {\mathrm{\texteuro}{13,467}}\: \hfill \\ \end{gathered} $$

Eq. 2: Calculation of the pharmacoeconomic benefit.

**Fig. 3:**
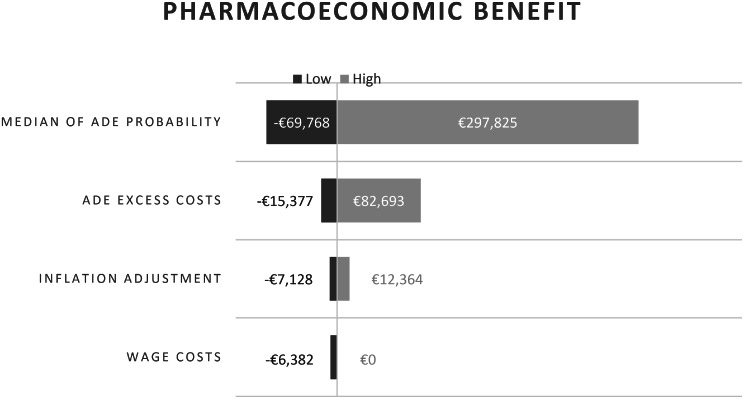
Deterministic sensitivity analysis about pharmacoeconomic benefit using a tornado diagram

The results of the deterministic sensitivity analyses are visualized in a tornado diagram (Fig. 3). Uncertainty was primarily driven by parameters relating to median ADE probabilities and associated excess costs. Each bar represents the impact of varying one parameter within its predefined range while holding all other parameters constant.

Simulating a further worst-case and best-case scenario determined a loss of -€64,190 and a gain of €706,317.

## Discussion

The role of CPs is essential in enhancing patient-specific medication through effective dosage recommendations [14]. In our study, these represented more than one quarter of the interventions. The acceptance rate of 52.6% is rather low compared with other international studies, e.g., 92% in the United States [15], 83% in Switzerland [16] or 60% in the Netherlands [17]. A recent meta-analysis of 15 randomised controlled trials reported that CP interventions reduced the ADE risk by approximately 14% (relative risk = 0.86) compared to usual care [18]. Our data corresponds to a rate of 17%, which aligns with literature. CP intervention savings are at the lower end of published data, which ranges from €20,000 to €50,000 (cost–benefit ratios: €3.3–€8.5 per €1 investment) [7, 19]. Our conservative assumptions are reflected in different variables: The inflation-adjusted, avoided ADE excess costs, adapted from Rottenkolber et al. (€1,211 in 2021) [8] are lower than e.g. Hoonhout et al. (€2,507 in 2010) [6]. Further examples are the more detailed division and a maximum value of an ADE probability of occurrence of 50% by Patanwala et al. [10] instead of 60% by Nesbit et al. [9], as well as the cautious expert evaluation of ADE occurrence. Although our expert panel could select an ADE probability occurrence score of up to 50%, a maximum range between 17.5% and 37.5% was used. In comparison, Liebing et al. assessed a single case with an ADE probability occurrence score of 60%, corresponding with avoided ADE additional costs of €1,953 [20]. They generated annual savings of €80,000 [20], indicating the higher the ADE probability occurrence score, the greater the pharmacoeconomic benefit.

Furthermore, we adjusted cost estimates using sector-specific medical inflation [11]. Applying an annual average rate of 2.5%, reflecting healthcare cost trends rather than relying solely on the general consumer price index. Results were sensitive to the parameter assumptions derived from heterogeneous sources, such as the expert panel or literature research. In this context, the heterogeneous composition of our expert panel was intended to represent a broader approach and minimize biases. Despite our conservative assumption, a positive pharmacoeconomic net benefit per FTE was determined. Sensitivity analyses demonstrated robustness of the economic results across a wide range of plausible assumptions. The best-case scenario indicates a pharmacoeconomic benefit that is approximately ten times higher than the loss in the worst-case scenario. Our findings are not widely generizable, as the analysis is based on a single tertiary care centre. The extent of the effects observed in other hospitals may be influenced by contextual factors such as staffing structures, the specific scope of CPS (e.g., the frequency of ward visits and degree of integration) and local prescribing practices. Furthermore, differences between healthcare systems, including reimbursement models such as case-mix and regulatory frameworks, may affect the implementation and financing of similar interventions.

Accordingly, our study suggests that CPs represent a worthwhile investment for avoiding ADE-related costs during hospitalization. Nevertheless, future research should consider findings with alternative models, such as cost-benefit ratio and return on invest analyses, to provide a broader understanding of potential outcomes [21]. Additionally, multicentre studies across diverse institutional and healthcare system contexts are needed to more robustly assess external validity.

## Limitations

This study faces some limitations. First, owing to our recommendation, the substituted medication was not recorded in the database, even though it was known. As a result, 29 interventions had to be excluded prior to the expert evaluation. This limitation was adjusted for improved traceability in future documentation. Second, despite shedding light on CP interventions, the single-centre design limits generalizability of our results. Third, the excess costs used in the preliminary ADE cost analysis were calculated from the hospital’s perspective [8]. Pharmaceutical interventions may also be associated with cost savings from a societal perspective. However, this issue was not taken into consideration. Finally, cost-avoidance analyses were based on hypothetical scenarios and do not represent actual ADE-related expenditure. This is due to the inherent uncertainty of cost-avoidance estimates. To address one part of this limitation, ADE excess costs determined in a large study applying propensity score matching [8] were used.

## Conclusion

Engaging clinical pharmacists in interventions contributes to optimized medication and improves economic outcomes. Although challenges remain in quantifying benefits, our approach results in a positive annual net benefit of €13,467 per FTE of a CP. The focus on collaborative care models highlights the potential for advancing patient-centred healthcare.

## Data Availability

Data supporting the findings of this study is not openly available due to reasons of sensitivity. Upon reasonable request, we are pleased to provide our data.

## Abbreviations

ADE: Adverse drug event
CP: Clinical pharmacist
CPS: Clinical pharmacy service
DRG: Diagnosis-related group
FTE: Full-time equivalent
